# Treatment of Vitamin D Deficiency in Decompensated Patients with Cirrhosis Is Associated with Improvement in Frailty

**DOI:** 10.3390/medsci13010030

**Published:** 2025-03-13

**Authors:** Raquel Díaz-Ruíz, Maria Poca, Eva Román, Berta Cuyàs, Irene Bañares, Ángela Morales, Elvira Hernández Martínez-Esparza, Rocío Panadero, Cristina Velasco, Marta Rapado-Castro, Irene Bretón, Rafael Bañares, German Soriano, Rita García-Martínez

**Affiliations:** 1 Department of Gastroenterology, Hospital General Universitario Gregorio Marañón, Facultad de Medicina, Universidad Complutense de Madrid, CIBERehd, 28007 Madrid, Spain; diaz.ruiz.r@gmail.com (R.D.-R.); rbanares@ucm.es (R.B.); 2 Instituto de Investigación Sanitaria Gregorio Marañón (IiSGM), 28007 Madrid, Spain; banares.irene@googlemail.com (I.B.); r.panaderogomez@gmail.com (R.P.); mrapado@iisgm.com (M.R.-C.); 3 Department of Gastroenterology, Hospital de la Santa Creu i Sant Pau, Institut de Recerca Sant Pau (IR Sant Pau), Universitat Autònoma de Barcelona, CIBERehd, 08025 Barcelona, Spain; mpoca@santpau.cat (M.P.); eroman@santpau.cat (E.R.); bcuyas@santpau.cat (B.C.); 4 University Nursing School EUI-Sant Pau, Hospital de la Santa Creu i Sant Pau, Universitat Autònoma de Barcelona, 08025 Barcelona, Spain; ehernandezma@santpau.cat; 5 Unidad de Nutrición Clínica y Dietética, Hospital General Universitario Gregorio Marañón, 28007 Madrid, Spain; apmoralesc@gmail.com (Á.M.); cvelascog@salud.madrid.org (C.V.); irenebreton@gmail.com (I.B.); 6 Department of Child and Adolescent Psychiatry, Institute of Psychiatry and Mental Health, Hospital General Universitario Gregorio Marañón, CIBERSAM, ISCIII, School of Medicine, Universidad Complutense, 28007 Madrid, Spain; 7 Department of Psychiatry, The University of Melbourne, Melbourne, VIC 3010, Australia; 8 Department of Internal Medicine, Hospital General Universitario Gregorio Marañón, Universidad Complutense de Madrid, CIBERehd, 28007 Madrid, Spain

**Keywords:** human, cirrhosis, frailty, sarcopenia, vitamin D

## Abstract

**Background/aim**: Frailty is increasingly recognized as a relevant prognostic factor in patients with cirrhosis, regardless of liver failure. Vitamin D deficiency is frequent in these patients and has been related to frailty and sarcopenia, but the impact of its supplementation on frailty in cirrhosis is unknown. The aim was to evaluate the effect of vitamin D supplementation on frailty in patients with decompensated cirrhosis and vitamin D deficiency or insufficiency. **Methods**: We included patients with cirrhosis who had vitamin D deficiency or insufficiency following their hospitalization for acute decompensation. Vitamin D was supplemented according to current recommendations, as were other micronutrients if necessary. Patients were followed for one year to evaluate changes at 6 and 12 months in frailty (Fried frailty index), health-related quality of life (SF-36, CLDQ) and mood (HADS). Body composition was assessed by DXA at baseline and at 12 months. **Results**: We included 39 patients, 27 of whom reached the 6-month follow-up. Serum vitamin D increased at 6 and 12 months (*p* < 0.001 compared to baseline). Fried frailty index improved at the 6-month visit (*p* = 0.004), and handgrip strength improved at 6 (*p* = 0.001) and 12 (*p* = 0.002) months, similarly in women and men. At 12 months, we observed an increase in body mass index, right arm lean mass and total fat mass. **Conclusions**: A multifactorial nutritional intervention, especially vitamin D supplementation after discharge in decompensated, vitamin D-deficient patients with cirrhosis, was associated with an improvement in frailty, muscular strength and lean muscle mass. However, the increase in fat mass strengthens the recommendation for diet, exercise and weight supervision.

## 1. Introduction

Frailty is increasingly recognized as a relevant prognostic factor in patients with cirrhosis, independently of the degree of liver failure as measured by conventional scoring systems such as Child–Pugh or MELD [1,2,3]. A mainstay component of the frailty syndrome in cirrhosis is sarcopenia, defined as a loss of muscle function and mass, but the concept of frailty is multidimensional and multifactorial [1,2,3]. Many factors are known to contribute to frailty in these patients, including vitamin deficits, especially vitamin D [4].

Vitamin D has gained interest in recent decades because of its pleiotropic extraskeletal functions [5], such as muscle function [6]. Its deficiency is a relevant finding in several diseases, including cardiovascular diseases [7], HIV infection [8], chronic hepatitis B [9] and sepsis-associated acute kidney injury [10]. In patients with cirrhosis, vitamin D hypovitaminosis has been associated with sarcopenia [4] and frailty [3]. The effect of vitamin D supplementation on muscle mass and strength has been evaluated in a previous study [11], but to our knowledge, the impact of vitamin D supplementation on frailty in patients with cirrhosis has not been specifically addressed.

The aim of this study was to assess the impact of vitamin D supplementation on frailty and body composition in vitamin D-deficient subjects with decompensated cirrhosis.

## 2. Materials and Methods

### 2.1. Design

This observational prospective study was performed in patients with cirrhosis and vitamin D deficiency who were discharged after hospitalization for decompensation at two tertiary hospitals in Spain (Hospital General Universitario Gregorio Marañón [HGUGM], Madrid and Hospital de la Santa Creu i Sant Pau [HSCSP], Barcelona). HGUGM performs liver transplants, but HSCSP does not. Consecutive patients discharged from the Hepatology Unit at either hospital between September 2017 and January 2020 were assessed before initiating vitamin D supplementation and at 6 and 12 months after enrolment.

### 2.2. Patients

Patients aged over 18 years and discharged after hospitalization for decompensated cirrhosis in the previous 6 weeks were screened for participation in the study. Exclusion criteria were: (i) human immunodeficiency virus infection; (ii) hepatocellular carcinoma beyond Milan criteria; (iii) comorbidities with life expectancy < 6 months; (iv) vitamin D supplementation at inclusion; (v) contraindication for vitamin D supplementation; (vi) neurological or musculoskeletal conditions that did not allow assessment; (vii) previous liver transplantation; (viii) recent (last 6 months) harmful use of alcohol (men > 40 g/d, women > 20 g/d); (ix) cholestatic liver disease such as primary biliary cholangitis or primary sclerosing cholangitis in order to avoid the contribution of impaired absorption of vitamin D by cholestasis; (x) hepatitis C antiviral therapy within the last 6 months; (xi) MELD > 30 at inclusion; and (xii) immunosuppressive therapy to avoid its potential effect on frailty and bone loss. A history of liver disease, complications of cirrhosis, comorbidities and medical treatment were recorded at baseline and during follow-up.

### 2.3. Treatment

All participants were evaluated following standard procedures. Vitamin D status was determined by measuring plasma levels of 25OHD in accordance with the local laboratory procedure. Patients with insufficiency (25OHD 20–30 ng/mL or 50–75 nmol/L) or deficiency (25OHD < 20 ng/mL or <50 nmol/L) were prescribed vitamin D supplements according to local guidelines available at the time of inclusion in the study, and supervised by the nutritionists. Briefly, patients with an insufficiency received 16,000 UI every two weeks, and those with a deficiency received 16,000 UI every week—all of them with regular controls and adjustments [12,13] (Supplementary Table S1). If any other nutritional deficits were detected, they were also supplemented by the physician in charge.

### 2.4. Anthropometric Assessments

We determined weight, height, body mass index and right arm measurements. Measurements in the arm consisted of mid-arm circumference and triceps skinfold thickness, both used to calculate mid-arm muscle circumference [14] and to estimate muscle mass and fat mass.

### 2.5. Frailty

Frailty was assessed according to the five criteria of the Fried frailty index (unintentional weight loss, reduced handgrip strength, slow walking speed, self-reported exhaustion and low physical activity). Patients were considered frail if they met ≥3 criteria and non-frail if they met 0–2 criteria (including pre-frail 1–2 criteria and robust 0 criteria) [3,15].

The Braden scale was used to evaluate pressure ulcer risk, and it was included as a complementary evaluation of frailty. This scale includes six domains: sensory perception, activity, mobility, moisture, nutrition, friction and shear. Higher scores imply lower risk [16].

We used the Fracture Risk Assessment Tool (FRAX) to calculate the percentage risk at 10 years of osteoporotic fracture and hip fracture on the basis of demographic and clinical characteristics of patients and femoral neck bone mineral density [17]. The Tinetti scale was used to evaluate balance, gait and the risk of falls. Higher scores indicate lower risk of fall [18].

### 2.6. Incidence of Falls and Other Clinical Events

Falls were assessed retrospectively and prospectively at each visit during the study using a previously described specific questionnaire and a review of clinical records [19]. The mean number of falls per month was calculated for the previous year and during follow-up. We also recorded hospitalizations, mortality and the need for liver transplantation during follow-up.

### 2.7. Handgrip Muscular Strength

Handgrip strength was evaluated using a dynamometer (KERN MAP-BA-s-0910) following the manufacturer’s instructions. We calculated the mean of three consecutive measurements. Values below 27 kg in men and 16 kg in women were used for the diagnosis of sarcopenia [20].

### 2.8. Body Composition by Dual Energy X-Ray Absorptiometry (DXA)

Body composition analysis was performed at baseline and 12 months by DXA with an iDXA scanner, GE Healthcare (Madison, WI, USA) in HGUGM and a Hologic Discovery DXA system (Hologic, Bedford, MA, USA) in HSCSP. The coefficient of variation was 1%. Scan acquisition and analysis were performed blindly by certified and experienced technicians in accordance with ISCD standards (https://iscd.org/, accessed on 1 January 2017). To avoid the potential confounding effect of leg oedema, we estimated the appendicular lean mass using only the lean mass of the arms.

### 2.9. Health-Related Quality of Life (HRQoL) and Mood: SF-36, CLDQ and HADS

The Medical Outcomes Short Form (SF-36) questionnaire was administered to patients to evaluate HRQoL. This test has 36 items that cover eight domains. The eight domains can be grouped into two measures: the Physical Component Score (including physical functioning, role-limitation physical, bodily pain and general health) and the Mental Component Score (vitality, social functioning, role-limitation emotional and mental health). Higher scores indicate better HRQoL, and the SF-36 has been validated for the Spanish population [21].

The Chronic Liver Disease Questionnaire (CLDQ) was also used to measure HRQoL. This tool includes 29 items distributed into six domains: fatigue, emotional function, worry, activity, abdominal symptoms and systemic symptoms. Each domain score is the mean of the items contained, and the overall score is the mean of all domains. Higher scores indicate better HRQoL, and CLDQ has also been validated for the Spanish population [22].

The Hospital Depression and Anxiety Scale (HADS) was used to evaluate mood in two subscales: depression and anxiety. Higher values indicate higher levels of depression or anxiety [23].

### 2.10. Analytical Determinations

A battery of analytical determinations, including parameters of nutrition, vitamins and oligoelements was performed at baseline, 6 months and 12 months.

### 2.11. Statistical Analysis

Results are expressed as the number of patients, percentages and median (inter-quartile range) or mean ± SEM. A Chi-square test or the Fisher exact test was used to analyse differences between categorical variables. The Student’s *t*-test or the Mann–Whitney test was used to compare continuous variables at baseline. Depending on the variable distribution, parametric or non-parametric tests were applied to study the changes in the variables using the paired Student’s *t*-test or the Wilcoxon test. The normality of continuous variables was evaluated using the Shapiro–Wilk test. Pearson’s or Spearman’s correlation coefficient test was applied to study correlations between variables. The Jonckheere–Terpstra test was applied to evaluate the association between quantitative variables organized in ordinal categories. A two-sided *p*-value < 0.05 was considered statistically significant. Statistical analysis was performed using IBM SPSS Statistics for Windows (version 29.0.1.1; IBM Corp, Armonk, NY, USA).

## 3. Results

**Figure 1:**
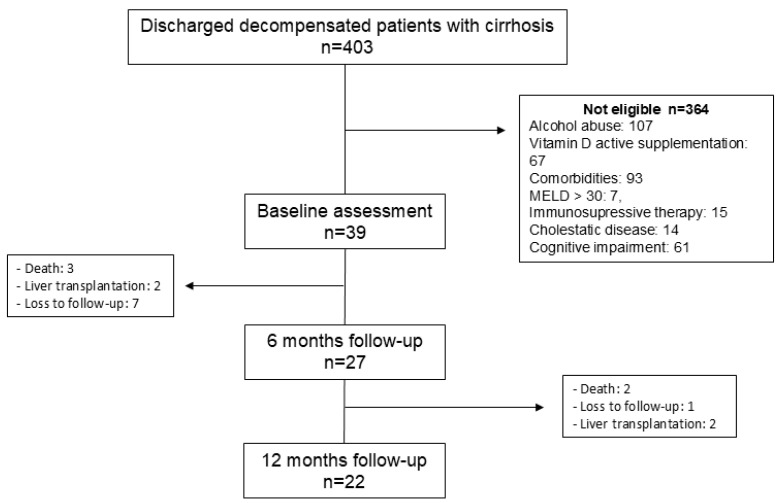
Flowchart of the study.

During the inclusion period, 403 patients were evaluated, and those meeting the inclusion criteria were invited to participate. A total of 364 were not eligible, and 39 were finally included (Figure 1).

Twenty-seven patients completed the 6-month evaluation (HGUGM = 14, HSCSP = 13) and were included in the analysis. Twenty-two patients completed the 12-month evaluation. Table 1 shows the characteristics of patients at inclusion. All patients presented vitamin D insufficiency (30%) or deficiency (70%). Patients from the HGUGM were younger and had more advanced liver insufficiency and lower right arm skinfold than patients from the HSCSP, probably because the HGUGM performs liver transplantation while HSCSP does not and refers candidates to liver transplantation to other centres. Patients from the HSCSP had higher FRAX scores, lower HADS depression and lower vitamin D levels than patients from HGUGM, probably because the patients were older in HSCSP. We did not find a correlation between the Fried frailty index or handgrip strength and vitamin D levels at baseline. The Fried frailty index, handgrip strength and vitamin D levels do not correlate with the liver function tests at baseline.

During follow-up, 5 of the 39 patients (12.8%) died, 11 (28.2%) required a total of 15 readmissions for decompensation of cirrhosis, 4 (10.2%) were transplanted and 8 (20.5%) dropped out. The causes of readmission were hepatic encephalopathy in 6 episodes, ascites in 5, hepatic encephalopathy and ascites in 3 and ascites and hepatorenal syndrome in one. One patient developed a de novo hepatocellular carcinoma. Seven patients (25.9%) presented falls during follow-up and two had fractures. The number of falls per patient/month during the previous year was 0.098 ± 0.047, and during the prospective follow-up, it was 0.043 ± 0.014, *p* = 0.56. Supplementary Table S2 shows supplementation of micronutrients other than vitamin D. Vitamin A was the most frequently supplemented (44.4% of patients at baseline, 59.3% at 6 months and 72.7% at 12 months), followed by iron and folic acid (22.2% and 14.8% at 6 months, respectively).

### 3.1. Frailty

**Figure 2:**
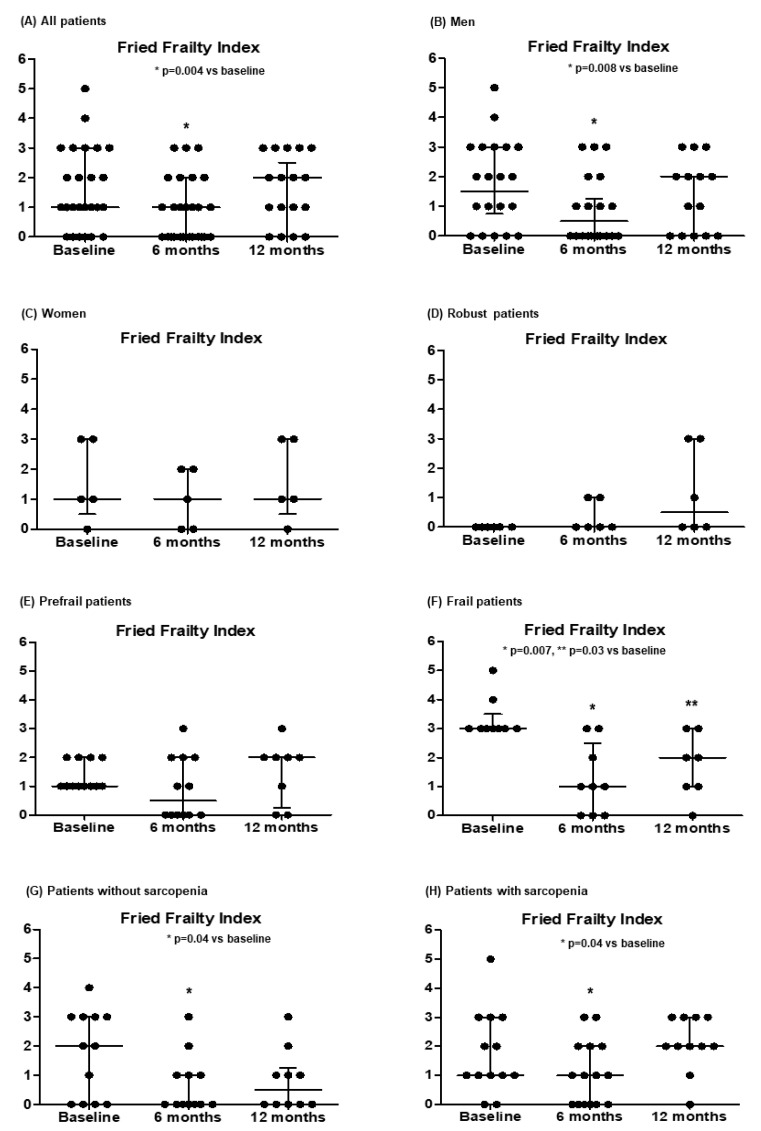
Evolution in the Fried frailty index in all patients (**A**) and separated according to sex (**B**,**C**), the degree of frailty (**D**–**F**) and the presence of sarcopenia (defined by a handgrip strength < 27 kg in men or <16 kg in women) (**G**,**H**) at baseline. Data are expressed as median and IQR.

Table 2 and Figure 2 show the changes in the parameters of frailty during the study. We observed an improvement in the Fried frailty index at 6-month visits, especially in men and frail patients. The nutrition domain of the Braden scale increased, reaching statistical significance at 12 months.

### 3.2. Handgrip Muscular Strength

**Figure 3:**
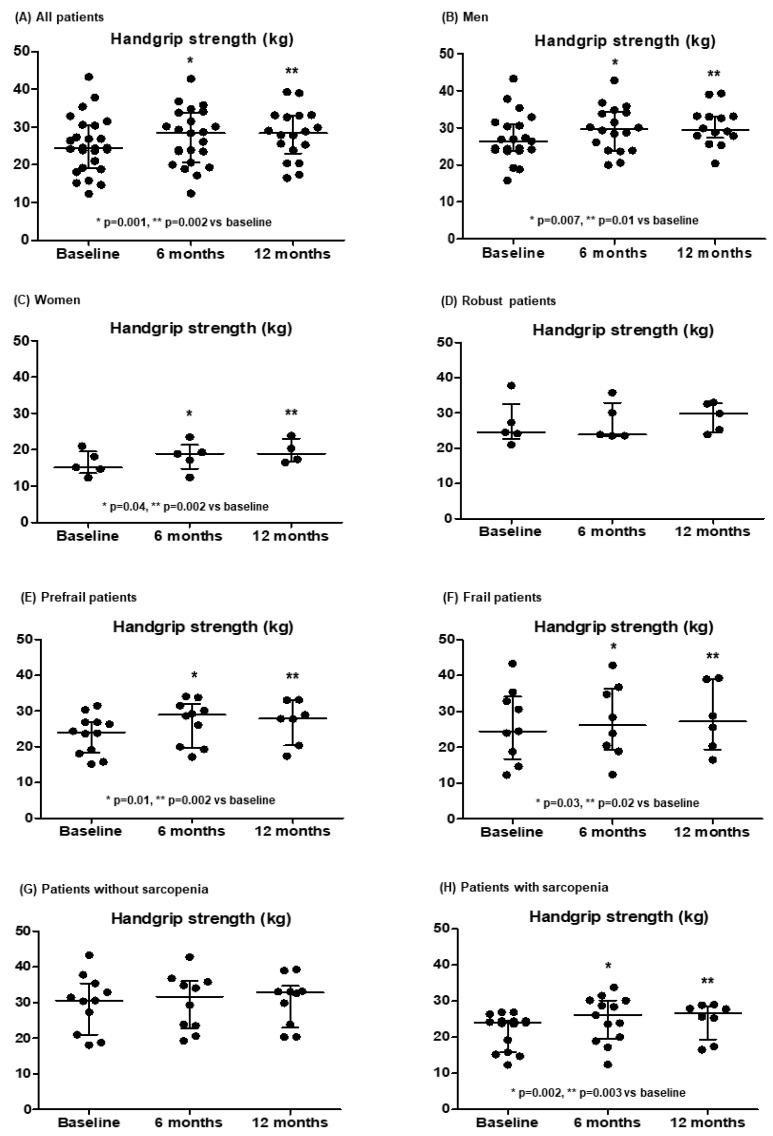
Evolution in the handgrip strength in all patients (**A**) and separated according to sex (**B**,**C**), the degree of frailty (**D**–**F**) and the presence of sarcopenia (defined by a handgrip strength < 27 kg in men or <16 kg in women) (**G**,**H**) at baseline. Results are expressed as median and IQR.

Table 2 and Figure 3 show the handgrip strength measures during the study. It improved significantly at 6 and 12 months, similarly in men and women, and especially in prefrail and frail patients and patients with sarcopenia.

### 3.3. Mood and HRQoL

Regarding mood and HRQoL, we observed an improvement in HADS anxiety, reaching statistical significance in frail patients at 6 months and in the mental component of the SF-36 questionnaire in frail and prefrail patients at 6 months.

### 3.4. Analytical Parameters

Regarding analytical parameters (Table 3), the main changes were an increase in haemoglobin, prealbumin, HDL cholesterol, vitamin D, vitamin A and folate, and a decrease in platelet count and parathyroid hormone. We did not observe significant changes in the liver function tests during the study. INR was the only liver function test whose change correlated with the change in the Fried frailty index or the change in the handgrip strength (r = 0.48, *p* = 0.03, and r = −0.50, *p* = 0.04, respectively, at 12 months). The change in vitamin D levels did not correlate with the change in any liver function test.

### 3.5. Body Composition

Table 4 shows the changes in body composition. Patients presented an increase in body mass index, right arm skinfold, lean right arm and body fat. Men with lower vitamin D levels at baseline showed a lower baseline right arm lean mass (Jonckheere-Terpstra *p* = 0.048) and a trend to a greater improvement in right arm lean mass at 12 months (Jonckheere-Terpstra *p* = 0.056). The increase in body mass index correlated with both the increase in total body fat (r = 0.537, *p* = 0.018) and the increase in total lean mass (r = 0.678, *p* = 0.002).

To appraise the potential contribution of spontaneous recovery after hospitalisation in the improvement of frailty and handgrip strength, we compared the change in these parameters between patients who required rehospitalisation during the 12 months of follow-up (n = 9) and those who did not (n = 13). We did not find significant differences between these two subgroups. The change in the Fried frailty index at 6 months was −0.60 ± 0.40 vs. −1.06 ± 0.31, and at 12 months, it was −0.12 ± 0.66 vs. −0.07 ± 0.46; the change in handgrip strength at 6 months was 2.96 ± 0.90 vs. 1.72 ± 0.77 kg, and at 12 months it was 3.37 ± 0.99 vs. 1.79 ± 0.81 kg, respectively. Trying to evaluate the potential contribution of supplements other than vitamin D, we compared the change in frailty and handgrip strength between patients receiving only vitamin D (n = 7) and those also receiving other supplements (n = 20) during the first 6 months. The change in Fried frailty index was −1.33 ± 0.42 vs. −0.75 ± 1.29, and the change in handgrip strength was 2.8 ± 1.47 vs. 2.1 ± 0.65, respectively (*p* not significant).

No significant correlations were observed between the change in vitamin D levels and the change in frailty and handgrip strength at 6 months (r = −0.06, *p* = 0.76; and r = −0.07, *p* = 0.75, respectively) or at 12 months (r = −0.10, *p* = 0.65; and r = −0.19, *p* = 0.45, respectively).

At 6 months, the Fried frailty index was 0 (0–1), and handgrip strength was 24 (20.3–30.8) kg in patients who showed sufficient vitamin D (>30 mg/mL) at this time point, and 1 (0–3) and 28.5 (22.7–36.1) kg, respectively, in non-sufficient patients (insufficient and deficient) (vitamin D ≤ 30 ng/mL). At 12 months, the Fried frailty index was 1 (0–2), and handgrip strength was 28.3 (21.2–33.1) kg in patients who were considered sufficient at this time point, and 2 (1–3) and 28.4 (24.1–32.2) kg, respectively, in non-sufficient patients. These differences did not reach statistical significance.

Because of the influence of sunlight exposure on serum vitamin D levels, we compared vitamin D levels at baseline between patients enrolled in spring or summer and patients enrolled in autumn or winter, and we observed a non-significant trend to higher levels in patients enrolled in spring or summer [17.2 (12.7–27.2) vs. 11.2 (8.2–14.6) ng/mL]. There were no statistically significant differences between these two groups regarding the changes in vitamin D, Fried frailty index or handgrip strength at 6 and 12 months with respect to baseline values (Supplementary Table S3).

## 4. Discussion

The main finding in this study was the improvement in frailty and handgrip strength following a multifactorial nutritional intervention that especially included vitamin D supplementation in decompensated patients with cirrhosis and vitamin D deficiency or insufficiency. To our knowledge, this is the first study to show an improvement in frailty after vitamin D supplementation in patients with cirrhosis.

Frailty is a multidimensional status that has been recognized as a relevant prognostic factor in several chronic diseases [15,18,20], including cirrhosis [1,2,3], independently of the degree of liver insufficiency. It has therefore been identified as a therapeutic target. Several strategies have been proposed to ameliorate frailty in these patients [1], such as exercise [24,25,26,27,28,29], nutritional interventions [4,28], branched-chain amino acid supplements [30,31], testosterone supplementation in men [32] and probiotics [33], alone or in combination [27,28,29,34]. Another measure that may potentially improve frailty in patients with cirrhosis is vitamin D supplementation because vitamin deficiencies, especially vitamin D, are frequent in patients with cirrhosis and have been related to frailty and sarcopenia [3,4,35].

In the present study, patients with cirrhosis and discharged after a hospitalization presented a severe deficiency in vitamin D at baseline, and most of them (78%) were frail or prefrail. After 6 months of vitamin D supplementation, we observed an improvement in frailty as assessed by the Fried frailty index. This index covers several domains related to frailty (unintentional weight loss, muscular strength, walking speed, exhaustion and physical activity) [15] and has been previously used in patients with cirrhosis [3]. Regarding the additional tools that we used to evaluate frailty, we observed a non-significant trend to improvement in the FRAX score that estimates the risk of fracture [17], in the Tinetti scale that evaluates balance, gait and risk of falls [18], and in the Braden scale that assesses the risk of pressure ulcers [16], with a statistically significant improvement in the nutritional domain of the Braden scale at 12 months. Although it could have been expected, to our knowledge, this is the first study to report an improvement in frailty after vitamin D supplementation in patients with cirrhosis. Interestingly, when analysing patients classified by sex and the degree of frailty, this improvement was statistically significant in men and in frail patients but not in women or in robust and prefrail patients. We do not know if these differences between women and men were due to the low number of women included in the study—most of the women who were considered for the study were excluded because they were already on vitamin D—or because the effect of vitamin D supplementation differs between sexes. Several studies have reported that the effects of vitamin D differ in men and women, specifically concerning frailty and sarcopenia, as recently reviewed [36]. To explain these differences, among other factors, women would be more vulnerable to vitamin D deficiency due to the increase in the expression of metabolising genes such as *Vdr*, *Cyp2r1* and *Cyp27b1* induced by oestrogens and because of their higher percentage of adipose tissue storing vitamin D. In line with our results, the relationship between vitamin D deficiency and muscle mass and strength seems to be stronger for men than for women [36].

Sarcopenia and decreased muscular function are considered a main feature of the frailty syndrome in patients with cirrhosis [1,3]. In this study, we observed a clear improvement in handgrip strength as an index of muscle function, both at 6 and 12 months and this was observed in both women and men. The increase in handgrip strength was more marked and statistically significant in frail and prefrail patients and in those with sarcopenia. Again, these subgroups of patients were those with more severely impaired muscle function, and therefore, they had a higher potential to improve. Regarding muscle mass evaluated by DXA, we observed an increase that reached statistical significance in the right arm. Interestingly, the group of patients with the lowest levels of vitamin D had the lowest right arm muscle mass at baseline and the greatest improvement in this parameter at 12 months. Our results are in line with the findings of Okubo et al. [11]. In a randomized clinical trial, they observed an improvement in muscle function and skeletal muscle mass index, evaluated by handgrip strength and DXA, respectively, after 12 months of vitamin D supplementation in patients with decompensated cirrhosis.

Our study was not designed to explore the mechanisms implicated in the improvement in muscle mass and function with vitamin D supplementation, but it has been reported that vitamin D regulates blood calcium levels and intracellular phosphate absorption (essential for muscle contractility), stimulates the expression of protein 1, which triggers the activation of myogenic determination factor 1 (MyoD1) leading to subsequent inhibition of myostatin, and enhances myoblast self-renewal and muscle protein synthesis [6].

In the study by Okubo et al., the body mass index and the percentage of body fat did not change after vitamin D supplementation, but it should be noted that the patients’ median body mass index at baseline was 22 kg/m^2^ [11]. In our study, most participants were overweight at baseline, and their body weight and fat body mass increased at 12 months. The relationship between vitamin D supplementation and body weight and fat remains controversial [37,38]. Some studies have shown vitamin D favours weight loss in obese people, while other studies have observed no effect [37,38]. An increase in body fat after vitamin D supplementation has also been reported [39]. Regarding the possible mechanisms to explain an increase in weight and body fat, it has been observed that vitamin D can enhance adipocyte differentiation and lipid accumulation [39]. Moreover, vitamin D deficiency has been associated with a decrease in appetite and weight loss in older adults [40]. In our study, the increase in the nutrition domain of the Braden scale could reflect an amelioration of appetite after vitamin D supplementation, and this could have contributed to the increase in weight, fat and lean mass. However, these increases cannot be attributed to vitamin D supplementation alone because the recovery itself after the baseline hospitalization probably played a relevant role. Independently of the mechanisms involved, these findings emphasize that together with the unavoidable supplementation of vitamin deficits in patients with cirrhosis, nutrition and exercise counselling are crucial to avoid an undesirable gain in fat body mass.

We were surprised by the lack of correlation between vitamin D levels and frailty and muscle function at baseline, as well as between changes in vitamin D and the changes in these parameters during follow-up. These results suggest that the relationship between vitamin D and frailty and muscle function is not lineal, as previously reported [41], and that it is vitamin D supplementation per se and not the precise levels reached that impact these parameters. Other potential explanations for these findings are the multifactorial nature of frailty and sarcopenia in cirrhosis, and the fact that patients also received other micronutrients in addition to vitamin D. The lack of correlation between the liver function tests and the Fried frailty index, handgrip strength and vitamin D levels emphasizes that frailty in patients with cirrhosis is not only related to the degree of liver insufficiency [1,2,3].

Also of note is the non-significant trend toward a lower number of falls per patient/month during follow-up compared to the previous year. As falls are a relevant clinical outcome related to frailty in patients with cirrhosis [3,19], their decrease during vitamin D supplementation could be a consequence of the improvement in frailty and muscle function. In effect, in the elderly, vitamin D deficiency has been associated with falls, and the risk of falling has been shown to decrease after vitamin D supplementation [6].

Regarding mood and HRQoL, the effects we observed were modest, but there was an improvement in HADS anxiety in frail patients and in the mental component of SF-36 in frail and prefrail patients. The relationship between impaired mood and vitamin D deficiency has been previously described [42].

The main changes in the analytical parameters were an increase in haemoglobin, prealbumin, HDL cholesterol, vitamin D, vitamin A and folate, probably reflecting a better nutritional status and vitamin supplementation. Among other beneficial metabolic effects, the increase in HDL cholesterol after vitamin D supplementation has been previously reported [43]. The decrease in platelet count at 12 months could be related to the progression of portal hypertension, which was not measured in the present study. Finally, a decrease in the parathyroid hormone could be expected with the increase in serum vitamin D levels [5].

Our study has several limitations. First, the small sample size and the low number of women included in the study did not allow a reliable analysis according to sex. However, patients were followed up for a relatively long period of time and evaluated using a comprehensive battery of clinical and analytical parameters. Second, this was not a randomized trial. The beneficial effects, mainly on frailty and muscle function, could therefore be due to causes other than vitamin D supplementation, such as spontaneous recovery after being discharged, but we could not include a control or placebo group in our patients with such a severe deficiency of vitamin D. Nevertheless, both those patients who required further hospitalisations during follow-up and those who did not presented a similar improvement in frailty and handgrip strength, suggesting vitamin D supplementation had a more relevant role in this improvement than recovery after hospital discharge. Another issue to consider is that the nutritional intervention did not consist of vitamin D supplementation alone because other micronutrients also related to frailty [44] were supplemented when indicated. However, patients receiving vitamin D only had a non-significant trend for an even greater improvement in frailty and handgrip strength than those also receiving other supplements. This finding would strengthen the role of vitamin D supplementation in the beneficial effects observed in both these parameters.

## 5. Conclusions

A multifactorial nutritional intervention, especially including vitamin D supplementation, was associated with an improvement in frailty, handgrip strength and lean mass in decompensated patients with cirrhosis and vitamin D deficiency or insufficiency. The concomitant increase in fat body mass outlines the need for a holistic assessment, with a special emphasis on nutritional and exercise counselling, in addition to vitamin supplementation in patients with cirrhosis.

## Figures and Tables

**Table 1:** Clinical and analytical characteristics of all patients and patients separated according to the centre of enrolment. Data are expressed as numbers, percentages and median (IQR). *p*-values in bold indicate statistically significant differences.

	All Patients n = 27	HGUGM n = 14	HSCSP n = 13	*p*-Value
Age (yr)	62 (55–72)	58 (51.5–64.7)	67 (56.5–76.5)	**0.02**
Gender (male; n, %)	22 (81.5%)	12 (85%)	10 (77%)	0.56
BMI (kg/m^2^)	28.7 (24.7–30.1)	28.6 (26.2–29.6)	28.7 (24.5–30.8)	0.59
Comorbidity (Charlson score)	6 (5–7)	6 (4.7–7.2)	6 (5–7)	0.72
**Treatments**				
Antidepressants (n, %)	1 (3.7%)	0 (0.0%)	1 (7.7%)	0.29
Benzodiazepines (n, %)	2 (7.4%)	0 (0%)	2 (15.4%)	0.12
Opioids (n, %)	1 (3.7%)	0 (0%)	1 (7.7%)	0.20
Diuretics (n, %)	19 (70.4%)	9 (64.3%)	10 (77%)	0.47
Beta-blockers (n, %)	16 (59.2%)	7 (50%)	9 (69%)	0.31
Non-absorbable disaccharides (n, %)	13 (48.1%)	6 (42.8%)	7 (53.8%)	0.70
Rifaximin (n, %)	5 (18.5%)	4 (28.6%)	1 (7.7%)	0.32
**Characteristics of cirrhosis**				
Alcohol aetiology (n, %)	9 (33.3%)	4 (28.6%)	5 (38.5%)	0.90
Child–Pugh score	7 (6–8)	7 (6–8.2)	7 (6–7)	0.17
Child–Pugh A/B/C (n, %)	9 (33%)/16 (59%)/2 (8%)	4 (29%)/8 (57%)/2 (14%)	5 (38%)/8 (62%)/0	0.15
MELD score	11 (9–14)	13 (10.7–17.5)	9 (7–11)	**<0.001**
Oesophageal varices (n, %)	21 (77.8%)	11 (78.6%)	10 (77%)	0.91
Hepatocellular carcinoma (n, %)	4 (14.8%)	3 (21.4%)	1 (7.7%)	0.31
Previous ascites (n, %)	24 (88.9%)	14 (100%)	10 (77%)	0.05
Previous variceal bleeding (n, %)	12 (44.4%)	7 (50%)	5 (38.5%)	0.54
Previous overt hepatic encephalopathy (n, %)	9 (33.3%)	6 (42.9%)	3 (23%)	0.27
Previous infection last year (n, %)	12 (44.4%)	9 (64%)	3 (23%)	0.054
Previous SBP (n, %)	7 (25.9%)	5 (36%)	2 (15.4%)	0.22
**Parameters of frailty and body composition**				
Fried frailty index	1 (1–3)	2 (1–3)	1 (0–3)	0.09
Handgrip strength (kg)	24.4 (19.1–0.5)	25.4 (22.6–30.8)	23.9 (16.6–27.2)	0.32
Previous falls/fractures (n, %) (previous year)	11 (41%)/2 (7.4%)	5 (36%)/1 (7.1%)	6 (46%)/1 (7.7%)	0.70/1.00
FRAX risk of osteoporotic fracture at 10 years (%)	3.7 (1.9–6.4)	1.9 (1.7–3.7)	5.4 (3.5–8.4)	**0.004**
FRAX risk of hip fracture at 10 years (%)	1.3 (0.3–2.3)	0.4 (0.2–1.6)	2 (0.6–3.1)	**0.03**
Right arm circumference (cm)	30 (25.7–32.4)	30.2 (25.8–32.2)	30 (24.5–33.5)	0.89
Right arm skinfold (mm)	1.4 (1.0–1.9)	1.1 (0.8–1.6)	1.6 (1.2–3.0)	**0.02**
Tinetti total score	27 (26–28)	27 (26.5–27.5)	28 (25.5–28)	0.45
Braden score	22 (22–23)	22 (21.7–23)	22 (22–23)	0.98
HADS anxiety score	5 (1–7)	5.5 (2.7–9)	2 (2–6.5)	0.10
HADS depression score	5 (2–8)	7 (4.7–9.2)	2 (0.5–5)	**0.001**
**Analytical parameters**				
INR	1.26 (1.18–1.45)	1.38 (1.21–1.58)	1.25 (1.12–1.27)	**0.006**
Bilirubin (mg/dL)	1.54 (1.27–2.40)	2 (1.07–3.4)	1.44 (1.33–1.55)	0.08
Albumin (g/L)	33.1 (30.2–39)	35.5 (28.7–40.5)	32 (31.5–35.4)	0.37
Sodium (mmol/L)	138 (136–140)	138 (135–141)	138 (136–139)	0.54
Creatinine (mg/dL)	0.81 (0.72–1.00)	0.87 (0.72–1.15)	0.78 (9.72–0.88)	0.20
25OH Vitamin D (ng/mL)	12.7 (8.8–21)	16.2 (12.4–24.9)	8.9 (5.5–12.2)	**0.046**

BMI—body mass index; MELD—Model for End-stage Liver Disease; FRAX—Fracture Risk Assessment Tool; HADS—Hospital Anxiety and Depression Scale.

**Table 2:** Evolution of the degree of frailty assessed by the Fried frailty index, handgrip strength, FRAX, Tinetti score, Braden scale, mood (HADS) and HRQoL (SF-36 and CLDQ) during the study. Data are expressed as number of patients and percentages or median (IQR). Numbers in bold indicate statistically significant differences.

	Baseline n = 27	6 Months n = 27	12 Months n = 22
Robust/prefrail/frail (n, %)	6 (22%)/12 (44%)/9 (33%)	**14 (52%)/10 (37%)/3 (11%) ^1^**	6 (27%)/10 (45%)/5 (23%) *
Handgrip strength (kg)	24.4 (19.1–30.4)	**28.4 (20.6–33.8) ^2^**	**28.3 (23–33) ^3^**
FRAX risk of osteoporotic fracture at 10 years (%)	3.7 (1.9–6.4)	-	3.3 (1.8–6.6)
FRAX risk of hip fracture at 10 years (%)	1.3 (0.3–2.3)	-	1.0 (0.2–2.6)
Tinetti total score	27 (26–28)	28 (26.5–28)	27.5 (22–28)
Braden scale total	22 (22–23)	22 (22–23)	23 (21–23)
- Braden scale nutrition	3 (3–4)	4 (3–4)	**4 (4–4) ^4^**
HADS anxiety			
- all patients (n = 27)	5 (1–7)	3 (1–6)	3 (1–7)
- frail patients (n = 9)	7 (3.5–11)	**3 (1–5.5) ^5^**	7 (3–9)
HADS depression	5 (2–8)	3 (0–6)	3 (0.7–5)
SF-36 physical component	42.1 (34.5–47.5)	40.5 (32.1–52.9)	46.1 (35.8–55.7)
SF-36 mental component			
- all patients	50.1 (41.5–54.7)	50.2 (43.5–56.3)	53.3 (46.1–58.7)
- frail and prefrail patients (n = 21)	45.3 (35.53.6)	**48.7 (40.6–53.2) ^6^**	51 (39–58.3)
CLDQ	5.03 (4.31–5.86)	4.93 (4.17–6.15)	5.24 (4.20–6.01)

FRAX—Fracture Risk Assessment Tool; HADS—Hospital Anxiety and Depression Scale; SF-36—Short Form Health Survey 36; CLDQ—Chronic Liver Disease Questionnaire. * Fried Frailty Index not available in one patient. ^1^ *p* = 0.04, ^2^ *p* = 0.001, ^3^ *p* = 0.002; ^4^ *p* = 0.02, ^5^ *p* = 0.01; ^6^ *p* = 0.02 with respect to baseline. *p* NS in the remaining comparisons.

**Table 3:** Evolution of the analytical parameters during the study. Data are expressed as numbers, percentages or median (IQR). Numbers in bold indicate statistically significant differences.

	Baseline n = 27	6 Months n = 27	12 Months n = 22
Haemoglobin (g/dL)	12.1 (9.9–13)	**13.3 (10.4–14.6) ^1^**	13.4 (10.8–14)
Platelets (1 × 10^9^/L)	85 (68–96)	83 (59–106)	**72 (60.5–85) ^2^**
Creatinine (mg/dL)	0.81 (0.72–1.00)	0.90 (0.75–1.12)	0.81 (0.73–1.05)
Sodium (mmol/L)	138 (136–140)	138 (136–141)	137 (135–140)
AST (U/L)	37 (29–48)	38 (31–55)	40 (31–64.5)
ALT (U/L)	25 (18–33)	33 (20–43)	28 (21–50.5)
ALP (U/L)	93 (50–195)	105 (49–251)	111 (66–199)
GGT (U/L)	134 (121–172)	140 (112–195)	130 (106–173)
INR	1.26 (1.18–1.45)	1.30 (1.14–1.42)	1.30 (1.20–1.48)
Bilirubin (mg/dL)	1.54 (1.27–2.40)	1.57 (1.00–2.40)	1.69 (1.11–2.81)
Albumin (g/L)	33.1 (30.2–39)	35.9 (30.3–40)	37.9 (31.9–39.1)
Child–Pugh score	7 (6–8)	7 (6–7)	7 (5–7.5)
Child–Pugh class A/B/C (%)	9 (33%)/16 (59%)/2 (8%)	13 (48%)/11 (41%)/3 (11%)	10 (45%)/9 (41%)/3 (14%)
MELD score	11 (9–14)	11 (9–14)	12 (10–15)
Prealbumin (mg/dL)	8.0 (6.0–11.0)	**10.0 (8.0–13.2) ^3^**	**11.0 (9.0–14.0) ^3^**
Triglycerides (mg/dL)	83.2 (61.1–114)	85 (69.8–123.2)	75.7 (66.5–178)
Cholesterol (mg/dL)	132 (92.5–153)	**164.5 (119.5–182.8) ^4^**	**154.3 (123.6–175.2) ^3^**
LDL cholesterol (mg/dL)	68 (44–98)	86.2 (61.2–110.2)	82.7 (66.9–98)
HDL cholesterol (mg/dL)	36.4 (24.4–45)	**47.4 (36.6–56.2) ^5^**	**48 (32–61.5) ^6^**
Magnesium (mg/dL)	1.8 (1.6–1.9)	1.8 (1.7–1.9)	1.8 (1.6–1.9)
Zinc (µg/L)	53.0 (45.8–66.0)	**66.4 (53.0–75.1) ^4^**	61.6 (44.6–68.6)
Copper (µg/L)	86 (78–100)	82 (66–102)	85 (69–98)
Ferritin (µg/L)	127 (38–272)	89 (29–313)	83 (44–177)
Calcium (mg/dL)	8.9 (8.6–9.6)	8.9 (8.7–9.4)	9.2 (8.9–9.5)
Phosphate (mg/dL)	3.4 (2.7–3.5)	3.4 (3.0–3.7)	3.2 (2.5–3.6)
25OH Vitamin D (ng/mL)	12.7 (8.8–21)	**37.2 (18.6–55.4) ^5^**	**44.5 (28.3–59.1) ^5^**
Vitamin D:			
Sufficient	0 (0%)	**17 (63%)**	**15 (68%)**
Insufficient	8 (30%)	**4 (15%)**	**5 (23%)**
Deficient (n, %)	19 (70%)	**6 (22%) ^5^**	**2 (9%) ^5^**
Parathyroid hormone (ng/L)	32.5 (28.3–53.0)	28.8 (17.0–51.8)	**25.1 (21.1–34.8) ^7^**
Vitamin A (µg/dL)	14 (10–22)	**24 (17–29) ^8^**	**23 (18–33) ^9^**
Vitamin E (µg/dL)	1102 (836–1387)	1148 (951–1319)	1038 (858–1537)
Vitamin B12 (ng/L)	677 (450–883)	634 (419–1221)	412 (337–721)
Folate (µg/L)	9.2 (6.6–16.1)	**12.7 (8.2–21.9) ^10^**	**15.2 (10.0–19.1) ^3^**

MELD—Model for End-stage Liver Disease. ^1^ *p* = 0.03; ^2^ *p* = 0.01; ^3^ *p* = 0.01; ^4^ *p* = 0.04; ^5^ *p* < 0.001; ^6^ *p* = 0.005, ^7^ *p* = 0.03; ^8^ *p* = 0.009; ^9^ *p* = 0.007; ^10^ *p* = 0.02 with respect to baseline.

**Table 4:** Changes in body composition during the study. Data are expressed as median (IQR). Numbers in bold indicate statistically significant differences.

	Baseline n = 27	6 Months n = 27	12 Months n = 22
**Anthropometry**			
BMI (kg/m^2^)	28.7 (24.7–30.1)	29.9 (25.5–30.1)	**29.8 (26.2–31.2) ^1^**
Right arm circumference (cm)	30 (25.7–32.4)	30.1 (28–33)	29 (26.5–32.5)
Right arm skinfold (mm)	1.43 (1.04–1.92)	**1.69 (1.11–2.50) ^2^**	1.73 (1.25–2.97)
Mid-arm muscle circumference (cm)	24.8 (20.9–28.1)	25.0 (22.3–26.5)	23.9 (21.1–25.4)
**DXA (n = 22)**			
Pelvis (g/cm^2^) median (IQR)	0.940 (0.877–1.074)	-	0.950 (0.847–1.098)
Spine (g/cm^2^)	0.937 (0.785–1.146)	-	0.968 (0.792–1.118)
Total bone (g/cm^2^)	1.077 (0.991–1.149)	-	1.067 (0.987–1.136)
Fat right arm (g)	974 (814–1386)	-	**1173 (932–1805) ^3^**
Fat right leg (g)	3168 (2421–3855)	-	**3487 (2890–4691) ^4^**
Fat trunk (g)	10,463 (7578–15,358)	-	**12** **,** **858 (9232–16** **,** **409) ^5^**
Android fat (g)	1613 (1239–2460)	-	**2073 (1482–2929) ^6^**
Total fat (g)	19,022 (16,763–27,243)	-	**25** **,** **889 (17** **,** **210–27** **,** **928) ^4^**
Fat (%)	26.2 (21.4–32.2)	-	**31.4 (25.5–36.2) ^7^**
Lean right arm (g)	2354 (2003–2884)	-	**2725 (2081–2988) ^1^**
Lean right leg (g)	7803 (6923–9058)	-	7815 (7077–8750)
Lean total (g)	52,774 (43,652–60,660)	-	54,181 (47,212–57,775)
Lean arms/height^2^ (g/m^2^)	1621 (1421–1832)	-	1805 (1437–1946) ^8^

BMI—body mass index; DXA—dual-energy X-ray absorptiometry. ^1^ *p* = 0.03; ^2^ *p* = 0.003; ^3^ *p* = 0.002; ^4^ *p* = 0.003; ^5^ *p* = 0.008; ^6^ *p* = 0.01; ^7^ *p* = 0.005; ^8^ *p* = 0.07 vs. baseline.

## Data Availability

The data that support the findings of this study are available from the corresponding author upon reasonable request.

## Supplementary Materials

The following supporting information can be downloaded at: https://www.mdpi.com/article/10.3390/medsci13010030/s1, Supplementary Table S1: Supplementation of vitamin D during the study; Supplementary Table S2: Supplementation of other micronutrients different from vitamin D during the study; Supplementary Table S3: Changes in vitamin D levels, Fried Frailty Index and handgrip strength at 6 and 12 months with respect to baseline in patients enrolled in autumn or winter and in spring or summer.

## References

[1] Tandon P., Montano-Loza A.J., Lai J.C., Dasarathy S., Merli M. (2021). Sarcopenia and frailty in decompensated cirrhosis. J. Hepatol..

[2] Wang S., Whitlock R., Xu C., Taneja S., Singh S., Abraldes J.G., Burak K.W., Bailey R.J., Lai J.C., Tandon P. (2022). Frailty is associated with increased risk of cirrhosis disease progression and death. Hepatology.

[3] Román E., Parramón M., Flavià M., Gely C., Poca M., Gallego A., Santesmases R., Hernández E., Nieto J.C., Urgell E. (2021). Frailty in outpatients with cirrhosis: A prospective observational study. Liver Int..

[4] Kappus M.R., Mendoza M.S., Nguyen D., Medici V., McClave S.A. (2016). Sarcopenia in Patients with Chronic Liver Disease: Can It Be Altered by Diet and Exercise?. Curr. Gastroenterol. Rep..

[5] Paul S., Kaushik R., Chawla P., Upadhyay S., Rawat D., Akhtar A. (2024). Vitamin-D as a multifunctional molecule for overall well-being: An integrative review. Clin. Nutr. ESPEN.

[6] Russo C., Santangelo R., Malaguarnera L. (2024). Valle MS. The “Sunshine Vitamin” and Its Antioxidant Benefits for Enhancing Muscle Function. Nutrients.

[7] Anilkumar S.A., Dutta S., Aboo S., Ismail A. (2024). Vitamin D as a modulator of molecular pathways involved in CVDs: Evidence from preclinical studies. Life Sci..

[8] Androutsakos T., Politou M., Boti S., Pittaras T., Kontos A., Kordossis T., Pouliakis A., Panayiotakopoulos G. (2024). Prevalence and Causes of Vitamin D Deficiency in a Cohort of Greek HIV-Infected Individuals: A Prospective, Single Centre, Observational Study. Curr. HIV Res..

[9] Wang P., Chen J., Li Z., Xiong H., Lei Z., Chen D., Zhang Y., Gao Z., Mo Z. (2024). Association of vitamin D with functional cure in chronic hepatitis B: Insights from a retrospective cohort study and an intervention study. Clin. Nutr. ESPEN.

[10] Chen L., Zhou T., Lv C., Ni H., Zhao Z., Zhou H., Hu X. (2024). Vitamin D supplementation can improve the 28-day mortality rate in patients with sepsis-associated acute kidney injury. Ren. Fail..

[11] Okubo T., Atsukawa M., Tsubota A., Ono H., Kawano T., Yoshida Y., Arai T., Hayama K., Itokawa N., Kondo C. (2021). Effect of Vitamin D Supplementation on Skeletal Muscle Volume and Strength in Patients with Decompensated Liver Cirrhosis Undergoing Branched Chain Amino Acids Supplementation: A Prospective, Randomized, Controlled Pilot Trial. Nutrients.

[12] Holick M.F., Binkley N.C., Bischoff-Ferrari H.A., Gordon C.M., Hanley D.A., Heaney R.P., Murad M.H., Weaver C.M. (2011). Evaluation, treatment, and prevention of vitamin D deficiency: An Endocrine Society clinical practice guideline. J. Clin. Endocrinol. Metab..

[13] Ross A.C., Taylor C.L., Yaktine A.L., Del Valle H.B., Institute of Medicine (US) Committee to Review Dietary Reference Intakes for Vitamin D and Calcium (2011). Dietary Reference Intakes for Calcium and Vitamin D.

[14] Wang J., Thornton J.C., Kolesnik S., Pierson R.N. (2000). Anthropometry in body composition. An overview. Ann. N. Y. Acad. Sci..

[15] Fried L.P., Tangen C.M., Walston J., Newman A.B., Hirsch C., Gottdiener J., Seeman T., Tracy R., Kop W.J., Burke G. (2001). Cardiovascular Health Study Collaborative Research Group. Frailty in older adults: Evidence for a phenotype. J. Gerontol. A. Biol. Sci. Med. Sci..

[16] Bergstrom N., Braden B.J., Laguzza A., Holman V. (1987). The Braden Scale for Predicting Pressure Sore Risk. Nurs. Res..

[17] Centre for Metabolic Bone Diseases UoSU (2020). FRAX WHO Fracture Risk Assessment Tool. https://www.sheffield.ac.uk/FRAX/.

[18] Kerr G.K., Worringham C.J., Cole M.H., Lacherez P.F., Wood J.M., Silburn P.A. (2010). Predictors of future falls in Parkinson disease. Neurology.

[19] Soriano G., Román E., Córdoba J., Torrens M., Poca M., Torras X., Villanueva C., Gich I.J., Vargas V., Guarner C. (2012). Cognitive dysfunction in cirrhosis is associated with falls: A prospective study. Hepatology.

[20] Cruz-Jentoft A.J., Bahat G., Bauer J., Boirie Y., Bruyère O., Cederholm T., Cooper C., Landi F., Rolland Y., Sayer A.A. (2019). Sarcopenia: Revised European consensus on definition and diagnosis. Age Ageing.

[21] Alonso J., Prieto L., Antó J.M. (1995). The Spanish version of the SF-36 Health Survey (the SF-36 health questionnaire): An instrument for measuring clinical results. Med. Clin..

[22] Ferrer M., Córdoba J., Garin O., Olivé G., Flavià M., Vargas V., Esteban R., Alonso J. (2006). Validity of the Spanish version of the Chronic Liver Disease Questionnaire (CLDQ) as a standard outcome for quality of life assessment. Liver Transpl..

[23] Herrmann C. (1997). International experiences with the Hospital Anxiety and Depression Scale--a review of validation data and clinical results. J. Psychosom. Res..

[24] Lai J.C., Dodge J.L., Kappus M.R., Wong R., Mohamad Y., Segev D.L., McAdams-DeMarco M. (2021). A multicenter pilot randomized clinical trial of a home-based exercise program for patients with cirrhosis: The Strength Training Intervention (STRIVE). Am. J. Gastroenterol..

[25] Jamali T., Raasikh T., Bustamante G., Sisson A., Tandon P., Duarte-Rojo A., Hernaez R. (2022). Outcomes of exercise interventions in patients with advanced liver disease: A systematic review of randomized clinical trials. Am. J. Gastroenterol..

[26] Williams F.R., Berzigotti A., Lord J.M., Lai J.C., Armstrong M.J. (2019). Review article: Impact of exercise on physical frailty in patients with chronic liver disease. Aliment. Pharmacol. Ther..

[27] Román E., Torrades M.T., Nadal M.J., Cárdenas G., Nieto J.C., Vidal S., Bascuñana H., Juárez C., Guarner C., Córdoba J. (2014). Randomized pilot study: Effects of an exercise programme and leucine supplementation in patients with cirrhosis. Dig. Dis. Sci..

[28] Chen H.W., Ferrando A., White M.G., Dennis R.A., Xie J., Pauly M., Park S., Bartter T., Dunn M.A., Ruiz-Margain A. (2020). Home-based physical activity and diet intervention to improve physical function in advanced liver disease: A randomized pilot trial. Dig. Dis. Sci..

[29] Hernández-Conde M., Llop E., Gómez-Pimpollo L., Fernández Carrillo C., Rodríguez L., Van Den Brule E., Perelló C., López-Gómez M., Abad J., Martínez-Porras J.L. (2021). Adding branched-chain amino acids to an enhanced standard-of-care treatment improves muscle mass of cirrhotic patients with sarcopenia: A placebo-controlled trial. Am. J. Gastroenterol..

[30] Colosimo S., Bertoli S., Saffioti F. (2023). Use of branched-chain amino acids as a potential treatment for improving nutrition-related outcomes in advanced chronic liver disease. Nutrients.

[31] Siramolpiwat S., Limthanetkul N., Pornthisarn B., Vilaichone R.K., Chonprasertsuk S., Bhanthumkomol P., Nunanan P., Issariyakulkarn N. (2023). Branched-chain amino acids supplementation improves liver frailty index in frail compensated cirrhotic patients: A randomized controlled trial. BMC Gastroenterol..

[32] Sinclair M., Grossmann M., Hoermann R., Angus P.W., Gow P.J. (2016). Testosterone therapy increases muscle mass in men with cirrhosis and low testosterone: A randomised controlled trial. J. Hepatol..

[33] Román E., Nieto J.C., Gely C., Vidal S., Pozuelo M., Poca M., Juárez C., Guarner C., Manichanh C., Soriano G. (2019). Effect of a multistrain probiotic on cognitive function and risk of falls in patients with cirrhosis: A randomized trial. Hepatol. Commun..

[34] Román E., Kaür N., Sánchez E., Poca M., Padrós J., Nadal M.J., Cuyàs B., Alvarado E., Vidal S., Ortiz M.À. (2024). Home exercise, branched-chain amino acids, and probiotics improve frailty in cirrhosis: A randomized clinical trial. Hepatol. Commun..

[35] Okubo T., Atsukawa M., Tsubota A., Yoshida Y., Arai T., Iwashita A.N., Itokawa N., Kondo C., Iwakiri K. (2020). Relationship between serum vitamin D level and sarcopenia in chronic liver disease. Hepatol. Res..

[36] Wierzbicka A., Oczkowicz M. (2022). Sex differences in vitamin D metabolism, serum levels and action. Br. J. Nutr..

[37] Karampela I., Sakelliou A., Vallianou N., Christodoulatos G.S., Magkos F., Dalamaga M. (2021). Vitamin D and Obesity: Current Evidence and Controversies. Curr. Obes. Rep..

[38] Chattranukulchai Shantavasinkul P., Nimitphong H. (2022). Vitamin D and Visceral Obesity in Humans: What Should Clinicians Know?. Nutrients.

[39] Wamberg L. (2013). Effects of vitamin D supplementation on body fat accumulation, inflammation, and metabolic risk factors in obese adults with low vitamin D levels—Results from a randomized trial. Eur. J. Intern. Med..

[40] Sripongpunku C., Aisawan P., Tanchanoj C., Thanapluetiwong S., Sriwannopas O., Chansirikarnjana S., Assavapokee T., Srisuwarn P., Ruangritchankul S. (2021). Factors associated with unintentional weight loss among older adults in a geriatric outpatient clinic of university hospital. PLoS ONE.

[41] Bischoff-Ferrari H.A., Giovannucci E., Willett W.C., Dietrich T., Dawson-Hughes B. (2006). Estimation of optimal serum concentrations of 25-hydroxyvitamin D for multiple health outcomes. Am. J. Clin. Nutr..

[42] Kouba B.R., Camargo A., Gil-Mohapel J., Rodrigues A.L.S. (2022). Molecular Basis Underlying the Therapeutic Potential of Vitamin D for the Treatment of Depression and Anxiety. Int. J. Mol. Sci..

[43] Zittermann A. (2009). Vitamin D supplementation enhances the beneficial effects of weight loss on cardiovascular disease risk markers. Am. J. Clin. Nutr..

[44] Lorenzo-López L., Maseda A., de Labra C., Regueiro-Folgueira L., Rodríguez-Villamil J.L., Millán-Calenti J.C. (2017). Nutritional determinants of frailty in older adults: A systematic review. BMC Geriatr..

