# Supplementary material for: Treatment of Vitamin D Deficiency in Decompensated Patients with Cirrhosis Is Associated with Improvement in Frailty
Source: Med Sci (Basel). 2025 Mar 13;13(1):30. doi: 10.3390/medsci13010030 (PMC11943887; doi:10.3390/medsci13010030)
Supplement: Supplementary file 1 [file medsci-13-00030-s001.zip › medsci-3407800-supplementary.pdf]

**Table S1**. Supplementation of vitamin D during the study.

| Baseline vitamin D status | Initial dose | Maintenance | Control | Objective |
|---|---|---|---|---|
| Insufficiency: 25OHD 20-30 ng/mL | Vitamin D 16000UI (0.266 mg) orally with meals /2 weeks | Vitamin D 16000UI (0.266 mg) orally with meals /2 weeks | 3 months | 25OHD 30-50 ng/mL Ideally 40ng/mL |
| Deficiency: 25OHD <20 ng/mL | Vitamin D 16000UI (0.266 mg) orally with meals /1 week | Vitamin D 16000UI (0.266 mg) orally with meals /1 week | 3 months | 25OHD 30-50 ng/mL Ideally 40ng/mL |

Dose adjustments during follow-up:

- If 25OHD < 60 ng/mL: initial dose was maintained.
- If 25OHD ≥ 60 ng/mL: Initial dose was reduced to the half of initial dose

**Table S2**. Supplementation of other micronutrients different from vitamin D during the study.

| | Baseline | 6 months | 12 months |
|---|---|---|---|
| Vitamin A (n, %) | 12 (44.4%) | 16 (59.3%) | 16 (72.7%) |
| Vitamin E (n, %) | 2 (7.4%) | 2 (7.4%) | 2 (9.1%) |
| Vitamin D (n, %) | 0 (0%) | 24 (88.8%) | 21 (95.4%) |
| Vitamin K (n, %) | 2 (7.4%) | 2 (7.41%) | 1 (4.5%) |
| Vitamin B12 (n, %) | 2 (7.4%) | 0 | 1 (4.5%) |
| Folic acid (n, %) | 3 (11.1%) | 4 (14.8%) | 1 (4.5%) |
| Calcium (n, %) | 2 (7.4%) | 2 (7.4%) | 2 (9.1%) |
| Magnesium (n, %) | 3 (11.1%) | 2 (7.4%) | 1 (4.5%) |
| Iron (n, %) | 5 (18.5%) | 6 (22.2%) | 3 (13.6%) |
| Protein supplements (n, %) | 2 (7.4%) | 2 (7,.4%) | 1 (4.5%) |
| Other (n, %) | 3 (11.1%) | 3 (11.1%) | 1 (4.5%) |

**Table S3**. Changes in vitamin D levels, Fried Frailty Index and handgrip strength at 6 and 12 months with respect to baseline in patients enrolled in autumn or winter and in spring or summer. Results expressed as median (IQR). No statistically significant differences between the two groups.

| | Autumn or winter n=16 | Spring or summer n=11 |
|---|---|---|
| Change in vitamin D levels at 6 months (ng/mL) | 25.1 (3.2-49.9) | 24.3 (-3.9-32.9) |
| Change in Fried Frailty Index at 6 months | -1 (-2-1) | -1 (-2-0) |
| Change in handgrip strength at 6 months (kg) | 1.8 (-0.2-4.6) | 2.0 (0.1-5.1) |
| Change in vitamin D levels at 12 months (ng/mL) | 27.2 (14.5-43.5) | 24.9 (1.5-48) |
| Change in Fried Frailty Index at 12 months | 0 (-2-1) | 1.5 (-2.2-3) |
| Change in handgrip strength at 12 months (kg) | 2.5 (1.5-4) | 2.2 (1.2-5.6) |
